# A new approach to OSCE preparation - PrOSCEs

**DOI:** 10.1186/s12909-019-1571-5

**Published:** 2019-05-02

**Authors:** James Bevan, Benjamin Russell, Ben Marshall

**Affiliations:** Faculty of Medicine, University of Southampton, Building 85, University Road, Southampton, SO17 1BJ UK

**Keywords:** OSCE, Revision, Medical education

## Abstract

**Background:**

Objectively structured clinical examinations (OSCEs) are a stressful experience for many health care students and professionals in training. Mock OSCEs have been shown to be beneficial for student OSCE preparation. However, due to their expense and administrative burden students may only get a few opportunities to partake in these. To address this gap in student preparation a series of regularly run totally peer led multi-role practice OSCEs (PrOSCEs) was developed.

**Methods:**

Fifteen PrOSCEs were run over five-months. A total of 32 second year medical students took part, all of whom were enrolled on the graduate-entry programme at the University of Southampton. In each PrOSCE, 18 participants rotated through the roles of ‘student’, ‘examiner’ and ‘patient’ in six simulated stations designed by their peers. Peer feedback was provided after each station. At the end of the series of PrOSCEs students were asked to fill in an anonymous online feedback survey to assess the usefulness of the PrOSCEs in exam preparation.

**Results:**

Twenty-two students responded to the survey. 100% of respondents deemed routine participation either ‘very useful’ or ‘useful’ in preparing for their exam. PrOSCEs were found to improve confidence (mean = 7.9/10, 95% CI 7.4–8.3), expected performance (mean = 7.5/10, 95% CI 6.8–8.2) and help guide revision (mean = 8.3/10, 95% CI 7.6–9.0). Self-perceived teaching performance and confidence in providing feedback was also positively associated with participation. The most beneficial roles were ‘student’ and ‘station creator’. Free-text feedback suggests that the informal setting and regular practice were particularly beneficial.

**Conclusion:**

The peer-led nature of the PrOSCEs allows for a low cost, low administrative burden and easy to replicate adjunct or alternative to large scale mock OSCEs. In addition the multi-role aspect of this approach could enhance exam preparation and may also improve aptitude as a clinical teacher. Further studies are required to understand if repeated practice has beneficial implications on OSCE performance.

## Background

Objectively structured clinical examinations (OSCEs) have become a source of dread for many in the allied health professions [1, 2]. Having been established as a reliable way to judge clinical aptitude and practical skills, OSCEs now are a mainstay of medical education and professional training where the format is used to ensure that students achieve minimum clinical standards. In the case of UK medical schools, a student’s performance in summative OSCEs often contributes a significant proportion to their final ranking upon graduation which influences their post-graduate employment opportunities.

Unlike written examinations, for which students can access practice questions relatively easily through online or faculty resources, OSCEs present a unique challenge to students as simulated OSCE practice is difficult to organise. Mock OSCEs run by university faculties and societies are aimed at ameliorating this challenge. However, these are often costly and are a significant administrative burden [3]. As such only a few mock OSCEs are made available to most students. Therefore, the majority of healthcare students will have completed just a small number of simulated OSCE stations before taking the real exam. This, combined with the often high-stakes nature of summative OSCEs, helps explain why these exams are recognised to be a source of anxiety and stress [4]. Indeed, a number of studies have found that practising OSCEs can lead to lower levels of anxiety and improved confidence [4–6].

In order to fill this gap in student preparation and given the well evidenced benefits of peer-to-peer teaching a peer-led strategy was implemented [6, 7]. As such a series of totally peer-led multi-role practice OSCEs (PrOSCEs) was developed.

The primary objective of running the PrOSCEs was to provide a low cost, low administrative burden format for OSCE practice. To evaluate students’ perceptions of the value of the format three different outcomes were assessed: whether the PrOSCEs were effective in guiding students’ revision, building confidence and improving expected performance in the students’ end of year summative OSCE (which are taken at the end of 3rd year for standard-entry medical students and at the end of 2nd year for graduate-entry medical students). Two further secondary outcomes were also assessed: whether the PrOSCEs improved students’ perceived aptitude in providing feedback and their confidence as a clinical teacher. While a number of studies have analysed the effects of singular mock OSCEs there is currently no literature assessing the effectiveness of regularly repeated, totally peer-led practice OSCEs.

## Methods

### The concept

The PrOSCEs were designed to closely replicate real OSCEs. In each PrOSCE all participants would spend one round acting as students in four consecutive OSCE-style stations under exam conditions and timings. Participants would also act as examiner and patient in two subsequent rounds. PrOSCEs were run regularly over a 5-month period to maximise student practice.

### Participant selection

The PrOSCEs were voluntary and made available to all 36 second year graduate-entry medical students at the University of Southampton through social media platforms and word of mouth. All students were resident at Basingstoke and North Hampshire Hospital undertaking placements in medicine, surgery and primary care. The students were all living together in hospital residence over this time period thus facilitating the logistics of the PrOSCEs.

Each PrOSCE required 18 participants. These were selected on a first-come-first-served basis through the utilisation of Facebook events to which all members of the cohort were invited. Participation once an individual’s place was confirmed was not obligatory but if the individual decided not to participate they were expected to find a replacement.

Additionally, as graduate entry medical students their relative maturity potentially enhanced participation and provision of feedback.

### Sharing administrative burden

Three days before each PrOSCE a request was sent out for 6 participants to volunteer to create a station: this involved preparing a brief for the ‘student’ and ‘patient’ and an objective mark scheme on which the ‘examiner’ could base their feedback. Participants were incentivised to create stations with a guaranteed place in the subsequent PrOSCE and were encouraged to create a station at least every third PrOSCE. This ensured that the administrative burden of creating stations was shared across the group over the 5 month period.

Station creators were requested to collaborate so that there was a mix of station types (i.e. history taking, examination, data interpretation and communication) and no overlap of topics in each PrOSCE. The station creation process was not formally regulated, but most participants tended to adapt their stations from freely-available online and library resources designed to help medical students practise for OSCEs.

### Running the PrOSCE

**Fig. 1:**
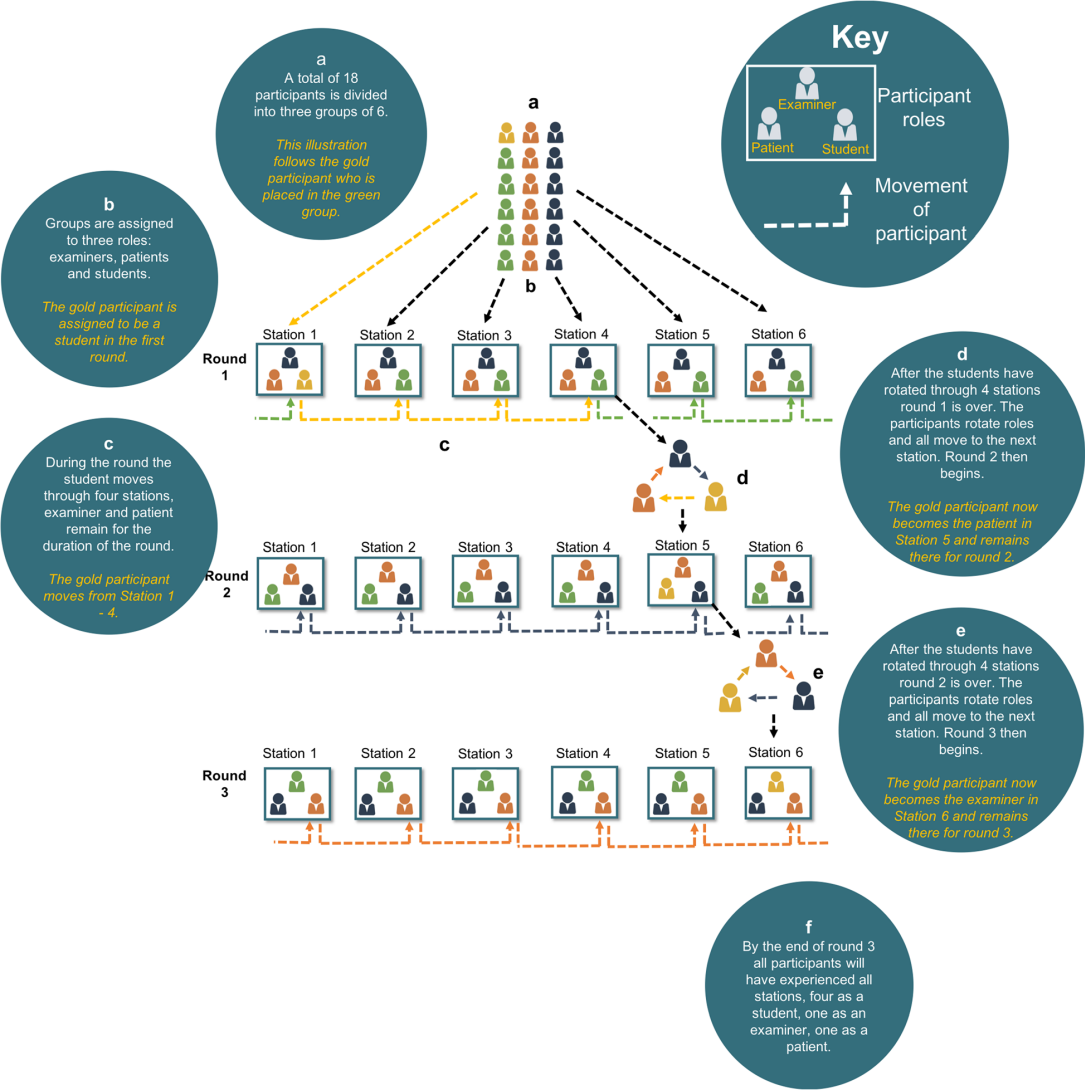
The process for each PrOSCE

The 18 participants were split, using a simple Microsoft Excel spreadsheet model, into three separate groups, assigned a role for the first round and given a starting station (see Fig. 1). Each station was filled by an examiner (in round 1, the station’s creators), a patient and a student which were randomly allocated. This schedule was posted on the Facebook event before the start of the PrOSCE so all participants knew where they started.

Each PrOSCE would run over three rounds timed to last for 4 stations. Participants assigned to be students for each round rotated through 4 stations, while examiners and patients remained in their assigned station for the duration of that round.

After each round, the groups would swap roles and moved to the following station to start the next round. Then the round would run as before. At the end of three rounds, all participants had experienced all 6 stations: 4 as a student, 1 as an examiner, and 1 as a patient.

Timings reflected the real OSCE: 90 s for students to read their brief and 8 min to complete the station (with a “1 minute remaining” warning). Unlike the real OSCE after each station there was 90 s for examiner feedback. Because feedback was not standardised, it was given in different formats. However, it was based on the mark scheme provided by the station creator.

At the end of each PrOSCE all participants would group in a communal area and station creators would give general feedback regarding their station. This was followed by a short group discussion of the topics covered during the PrOSCE where participants were encouraged to share specific ideas and concerns.

### Costs

The PrOSCEs were run in the hospital accommodation where the students were resident so no venue hire was required. The only cost incurred with running the PrOSCEs was that of printing mark schemes. This was the responsibility of the station creators and therefore was shared across the group over the 5 month period.

### Survey evaluation

Once the PrOSCE series had ended, participants were asked in the week preceding their summative OSCEs to respond to an anonymous survey with questions designed to evaluate the three primary outcomes and two secondary outcomes. Data analysis was performed on the survey results to identify trends in participants’ responses. Freidman analysis was conducted to analyse whether there was a particular station type or role which participants preferred. This was done using a free online tool (Vassarstats.net). Regression analysis was conducted using Microsoft Excel data analysis tools to explore whether there was a benefit to attending multiple PrOSCEs. For the purposes of analysis the mid-point was used for each option’s values (e.g. 5 was used for the person attending between 4 and 6 sessions). The free-text boxes in the survey were analysed to identify any particular trends in feedback, but no formal qualitative analysis was performed.

## Results

### Overview

A total of 15 PrOSCEs were run over a five-month period from February–June 2018. Out of the 36 participants invited to the PrOSCEs, 32 (89%) attended at least one PrOSCE. A total of 1080 stations were run over this period (72 stations in each of the 15 PrOSCEs).

The anonymised online survey was sent to all 32 participants once the PrOSCEs had finished and prior to the summative OSCE itself. 22 participants (69%) responded to the survey. Of these, 2 attended 1–3 sessions, 1 attended 4–6 sessions, 10 attended 7–9 sessions, 6 attended 10–12 sessions, and 3 attended 13–15 sessions.

### Survey results

The survey had 15 questions which assessed the participants’ perceptions relating to the primary and secondary outcomes and the quality of the PrOSCEs in general.

## Assessing outcomes

### Overall usefulness of the PrOSCEs

All respondents said that they found routine participation in the PrOSCEs useful: 81% found it “very useful”, and 19% found it “somewhat useful”, 0% found it “not useful” or “counterproductive”.

**Fig. 2:**
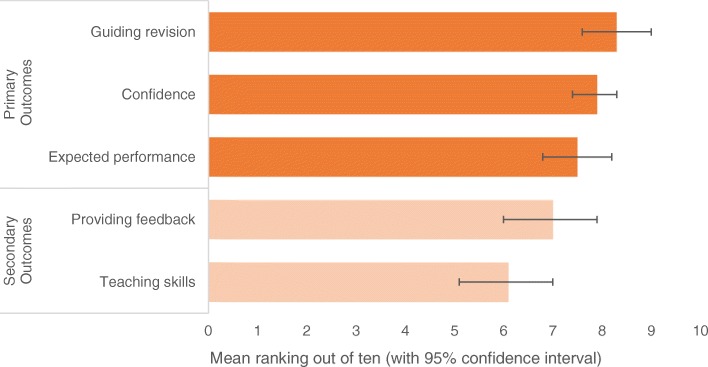
Participant ratings of PrOSCEs in terms of primary and secondary outcomes

Figure 2 summarises the survey results relating to ‘sliding-scale’ questions. Respondents ranked PrOSCEs particularly highly for guiding revision.

## Quality of the PrOSCEs

Participants rated the mean quality of the stations at 7.3 out of 10 (95% CI 6.6–8.1).

### Ranking usefulness

Tables 1 and 2 show the mean ranking in terms of perceived usefulness of each PrOSCE role and station type. ‘Student’ was the most useful role, and ‘history-taking stations’ were the most useful station type. Friedman analysis of the rankings confirms these rankings are statistically significant both for preferred roles [*χ*^2^ (3) = 23.29, P = < 0.001] and preferred station types [*χ*^2^ (3) = 27.00, P = < 0.0001].

**Table 1:** Mean usefulness ranking by role

Preferred role	Mean ranking (1 = Highest, 4 = Lowest)	Significance
Student	1.4	*P* = < 0.001
Creating a station	2.5	
Examiner	2.9	
Patient	3.1

**Table 2:** Mean usefulness ranking by station type

Station Type	Mean ranking (1 = Highest, 4 = Lowest)	Significance
Histories	1.5	*P* = < 0.0001
Communications	2.3	
Exams	2.6	
Data interpretation	3.5	

### Benefits of repeated attendance

The results of this are shown in Table 3. Repeated attendance was not found to be significantly correlated with either primary or secondary outcomes. This suggests that the perceived benefits of attending PrOSCEs were independent of the number attended.

**Table 3:** Regression analysis of benefits of repeated attendance

Outcome	Survey question topic	R^2^ value	*P* value
Primary	Guiding revision	0.001	0.89
Primary	Confidence	0.01	0.62
Primary	Expected performance	0.001	0.88
Secondary	Teaching skills	0.1	0.16
Secondary	Providing feedback	0.12	0.11

### Free-text positive feedback

Four themes emerged in the survey. The most commonly-cited benefits were the regular OSCE practice and the positive learning environment. The response below was typical:

*“I … enjoyed the opportunity to continuously work on OSCEs; it filled the gap between book-based learning and ward work.”*

Two other common themes were the variety of stations on offer and the immediate feedback provided by peers. The following two comments reflect these two themes:

*“it is a ‘safe’ environment in which to practice skills and learn from each other.”*

*“It was good to receive weekly feedback on my practical skills and made me feel more confident going into exams.”*

### Free-text suggestions for improvements

Although four respondents said that “nothing” was needed to improve the PrOSCEs, there were two common themes amongst those offering suggestions. The most common related to inconsistent and/or poor examiner feedback, for example:

*“[There should be] consistent mark schemes for stations (some were of poorer quality) and a short training session on how to give good feedback.”*

The second common theme was that respondents wanted the PrOSCEs to be more ‘realistic’. The response below was typical:

*“[There should be] more exam-like conditions - but I appreciate it is very hard to do.”*

## Discussion

This study demonstrates the value of the PrOSCE format as a tool for OSCE preparation. As well as providing a positive learning environment, the PrOSCE format shows that good-quality simulated OSCE preparation can be achieved without the high costs and administrative burden of large scale mock OSCEs. Indeed, there was a 100% pass rate amongst the PrOSCE participants in the summative OSCEs.

The totally peer-led aspects of the PrOSCE format was a strength of this approach. Spreading the workload of creating stations helped to reduce individual administrative burden. In addition the very act of creating stations was shown to have intrinsic value in itself; ‘station creator’ was considered the second most useful role. Free-text feedback and numerical ranking results indicated that participants were generally satisfied with station quality. This was despite there being no formal methodology to standardise the quality of stations and no means for survey respondents to know for certain that the quality was representative of the forthcoming summative exams. However, as all participants had undertaken a formal OSCE the previous year and had also participated in University mock OSCEs, they had a good sense of the standard expected. In addition, station creators made extensive use of OSCE revision resources (online and in print) to help guide them in creating realistic stations.

The role of ‘student’ was deemed the most useful and this was reassuring as the PrOSCEs were specifically designed to provide participants with an opportunity to practise being a student in an exam-like setting. While ranked lowest, the roles of ‘patient’ and ‘examiner’ have in the literature been shown to be beneficial to students, therefore implying that all roles in the PrOSCE are beneficial in OSCE preparation [8, 9].

The PrOSCEs were universally considered to be useful, and responses relating to the primary outcomes were ranked highly (guiding participants’ revision, improving confidence, and boosting expected performance). The secondary outcomes (providing feedback and developing teaching skills) were less highly rated by participants, which might be because these aspects are likely to be of lower priority for participants studying for exams. Future PrOSCEs should involve standardised participant feedback to enhance the learning experience and to help students develop this important clinical skill. To help address this, future PrOSCEs could recruit faculty members to run an initial teaching session on how to provide good quality feedback and create PrOSCE stations of comparable quality to the real exams.

The survey responses indicated that the perceived benefits of PrOSCEs were independent of the number of sessions attended. The analysis into this correlation, however, was limited by several factors. Survey respondents were asked to estimate the number of PrOSCEs they attended, which may have been inaccurate and as the responses were anonymised it was not possible to cross-reference this with participants’ actual attendance. The study had further limitations in that the data set was small and there was clustering of the data (most people attended between 7 and 11 PrOSCEs), meaning that it is difficult to accurately interpret the results of the regression analysis. In order to better investigate this possible correlation, future studies should recruit a larger number of participants and randomly allocate them to attend a fixed and differing number of sessions.

## Conclusions

PrOSCEs developed a positive environment in which to practise clinical skills. This format was shown to improve confidence, expected performance and guide revision in advance of a summative OSCE. Students had a very positive response to their attendance and all who partook in the PrOSCE series passed their end of year OSCE assessment.

This study was limited by the sample size. The results did not confirm any added benefit in attending repeated PrOSCEs: the benefit of participation appeared independent of the number of PrOSCEs attended. Further and larger studies would be needed in order to explore this correlation and the overall benefits of the PrOSCE format in more detail.

Despite this study’s limitations the PrOSCE format does represent an OSCE preparation approach that is cheap and easy to replicate. In this respect, the implementation of the PrOSCEs met the primary objective of creating a low cost, low administrative burden format for OSCE practice. PrOSCEs therefore have significant potential to help healthcare students or professionals in training gain confidence and guide revision in preparation for their exams.

## Abbreviations

OSCE: Objectively Structured Clinical Exam
PrOSCE: Practice OSCE
